# PARP Inhibitor PJ34 Protects Mitochondria and Induces DNA-Damage Mediated Apoptosis in Combination With Cisplatin or Temozolomide in B16F10 Melanoma Cells

**DOI:** 10.3389/fphys.2019.00538

**Published:** 2019-05-07

**Authors:** Anna Maria Cseh, Zsolt Fabian, Ruben Quintana-Cabrera, Aliz Szabo, Krisztian Eros, Maria Eugenia Soriano, Ferenc Gallyas, Luca Scorrano, Balazs Sumegi

**Affiliations:** ^1^Department of Biochemistry and Medical Chemistry, Medical School, University of Pécs, Pécs, Hungary; ^2^Department of Biology, University of Padova, Padua, Italy; ^3^Department of Medical Chemistry, Molecular Biology and Pathobiochemistry, Faculty of Medicine, Semmelweis University, Budapest, Hungary; ^4^Institute of Functional Biology and Genomics, University of Salamanca, Consejo Superior de Investigaciones Científicas, Salamanca, Spain; ^5^Institute of Biomedical Research of Salamanca, University Hospital of Salamanca, University of Salamanca, Consejo Superior de Investigaciones Científicas, Salamanca, Spain; ^6^CIBERFES, Instituto de Salud Carlos III, Madrid, Spain; ^7^Nuclear-Mitochondrial Interactions Research Group, Hungarian Academy of Sciences, Budapest, Hungary; ^8^Szentagothai Research Centre, University of Pécs, Pécs, Hungary; ^9^Venetian Institute of Molecular Medicine, Padua, Italy

**Keywords:** PARP, mitochondria, cancer, melanoma, cell death

## Abstract

PARP-1 inhibition has recently been employed in both mono- and combination therapies in various malignancies including melanoma with both promising and contradicting results reported. Although deeper understanding of the underlying molecular mechanisms may help improving clinical modalities, the complex cellular effects of PARP inhibitors make disentangling of the mechanisms involved in combination therapies difficult. Here, we used two cytostatic agents used in melanoma therapies in combination with PARP inhibition to have an insight into cellular events using the B16F10 melanoma model. We found that, when used in combination with cisplatin or temozolomide, pharmacologic blockade of PARP-1 by PJ34 augmented the DNA-damaging and cytotoxic effects of both alkylating compounds. Interestingly, however, this synergism unfolds relatively slowly and is preceded by molecular events that are traditionally believed to support cell survival including the stabilization of mitochondrial membrane potential and morphology. Our data indicate that the PARP inhibitor PJ34 has, apparently, opposing effects on the mitochondrial structure and cell survival. While, initially, it stimulates mitochondrial fusion and hyperpolarization, hallmarks of mitochondrial protection, it enhances the cytotoxic effects of alkylating agents at later stages. These findings may contribute to the optimization of PARP inhibitor-based antineoplastic modalities.

## Introduction

Poly(ADP-ribose) polymerase-1 (PARP-1) catalyzes the transfer of ADP-ribose from NAD^+^ to a wide range of target proteins leading to the formation of large, branching PAR chains that modulate enzymatic activity, DNA binding or regulatory properties in gene expression (Jiang et al., 2015; Gibson et al., 2016). PARP-1 also regulates gene expression by self-ADP-ribosylation-dependent binding to specific DNA sequences and by ADP-ribosylation of the PARP-1-CTCF-Dnmt1 complex that prevents DNA methylation (Zampieri et al., 2012; Gibson et al., 2016). Increased activation of PARP-1 in oxidative stress substantially reduces NAD^+^ levels that contribute to the compromised energy state and lead to necrotic cell death. In accordance, PARP inhibitors were found to have dramatic protective effects in several disease models where necrotic or apoptotic cell death is a key critical pathological factor (Pacher et al., 2002; Robaszkiewicz et al., 2012; Gero et al., 2014; Eros et al., 2017; Korkmaz-Icoz et al., 2018).

The PARP inhibitors were used successfully as monotherapy of BRCA1/2 mutated cancers, based on the fact that PARP inhibitions reduce the rate of DNA repair (Bhattacharjee and Nandi, 2017; Ashworth and Lord, 2018). PARP inhibitors were also found to facilitate the efficacy of both cytostatic agents like cisplatin and irradiation (Sakogawa et al., 2013; Bang et al., 2017; Litton et al., 2018). Considering that (i) PARP inhibitors were found to protect cells against oxidative stress-induced cell death and (ii) oxidative stress often accompanies radio- and chemotherapy, the molecular effects of PARP inhibitors in the distinct pathological situations remained obscure (McQuade et al., 2018).

Melanoma is one of the most malignant cancers harboring defects in repair and cell-cycle regulation. Indeed, it is believed that melanoma is associated with malfunctioning nucleotide excision repair and is among the most commonly reported cancers carrying *BRCA2* mutations (Breast Cancer Linkage Consortium, 1999; Di Lucca et al., 2009; Moran et al., 2012; Mersch et al., 2015). Because of their observed efficacy in BRCA-mutated tumors and the role of PARP-1 in cellular repair mechanisms, PARP inhibitors were introduced into melanoma therapy (Bryant et al., 2005). In melanomas, PARP inhibitors promoted cell death in combination with temozolomide both *in vitro* and in clinical studies but the underlying mechanism of action remains to be elucidated (Plummer et al., 2013; Gill et al., 2015; Middleton et al., 2015). To have a further insight into the role of PARP inhibition in combination therapy, we investigated the effects of the PARP inhibitor PJ34 when applied in combination with cisplatin or temozolomide using the B16F10 *in vitro* melanoma model. We found that PARP-inhibition exerts complex, apparently opposing, effects on cellular physiology. Indeed, while pharmacologic PARP-inhibition triggers mitochondrial processes that are known to be associated with cell survival, it also potentiates the cytotoxic effects of cytostatic compounds in B16F10 cells. This dichotomy may, at least in part, provide explanation to the controversial clinical observations upon the use of pharmacologic PARP inhibition as part of anti-neoplastic interventions.

## Materials and Methods

### Reagents

Chemicals were purchased from (Sigma-Aldrich S.r.l., Milan, Italy) unless otherwise stated. The PARP inhibitor compound PJ34, temozolomide and cisplatin were used at 10, 25, and 25 μM concentrations, respectively. The mitochondrial targeted dsRED (mtRFP) corresponding to pDsRed2-Mito and the pPARPGFPC1/N3 construct has been previously described in Cipolat et al. (2004) and Tapodi et al. (2005), respectively.

### Cells and Cell Cultures

Mouse B16F10 melanoma cell line was obtained from American Type Culture Collection (Manassas, VA, United States) and maintained in Dulbecco’s Modified Eagle Medium (Invitrogen, Life Technologies, Milan, Italy) supplemented with 10% (v/v) fetal bovine serum (Thermo Fisher, Life Technologies, Milan, Italy), 1% (v/v) penicillin/streptomycin and glutamine mixture (Invitrogen, Life Technologies, Milan, Italy). Transiently transfected B16F10 cells were generated using Transfectin Lipid Reagent (Bio-Rad Laboratories S.r.l., Milan, Italy) according to the manufacturer’s instructions. After 4–6 h of incubation, the medium was replaced to complete culture medium and the experiments were performed 24 h post-transfection.

### MTT Assay

Cells were seeded in flat-bottom 96-well plates at the 2.5 × 10^4^ per well density and cultured overnight before the assay. Following treatments, medium was replaced to a fresh one containing 0.5% 3-(4,5-dimethylthiazol-2-yl)-2,5-diphenyltetrazolium bromide (MTT) tetrazolium substrate and incubated for 3 h. The water-insoluble violet formazan precipitate was solubilized in 100 μl 20% sodium dodecyl sulfate solution and optical densities were measured by an Infinite 200 Pro plate reader (Tecan Italia S.r.l., Milan, Italy) at 570 nm. All experiments were run at least in four parallels and repeated three times.

### Clonogenic Cell Survival Assay

Cells were plated in 6-well plates at 300 cells/well density and cultured overnight before treatments and incubated for 10 days post-treatment. Following the incubation period, cells were washed with 1 × PBS and stained with 0.1% Coomassie blue (Bio-Rad Laboratories S.r.l., Milan, Italy) in 30% methanol and 10% acetic acid. Plates were scanned and the number of colonies was determined using the ImageJ software.

### Analysis of Cell Death

B16F10 cells were seeded into 6-well plates at a starting density of 2 × 10^4^cell/well and cultured for 24 h before treatments. Samples were stained with FITC-labeled Annexin-V and Propidium iodide (eBioscience, Life Technologies, Milan, Italy) according to the manufacturer’s protocol. Cell death was measured by flow cytometry using a FACS Calibur flow cytometer (Becton Dickinson Italia S.r.l., Milan, Italy), and the data were analyzed by CellQuest Pro software.

### Modified Alkaline Single Cell Gel Electrophoresis (Comet Assay)

Microscope slides were coated with a layer of 1% normal melting point agarose in PBS. B16F10 cells were seeded into 6-well plates at a starting density of 7 × 10^4^ cell/well. After treatment, cells were harvested, centrifuged, and mixed rapidly with 500 μl of 1% pre-warmed low melting point agarose in PBS. 50 μl suspension was pipetted onto the pre-coated slides and kept at 4°C for 10 min. Slides were incubated in lysis solution (2.5 M NaCl, 300 mM Tris, 200 mM NaOH, 3 mM Na2EDTA, 1% Triton X-100) for 15 min and electrophoresed at 25 V and 300 mA at 4°C for 30 min. Slides were neutralized three times for 5 min using Tris buffer (0.58 M, pH 7.5) and immersed in 70% ethanol for 5 min. Slides were stained with 0.25 μg/ml Hoechst 33342 for 10 min, washed two times in PBS and visualized by a Nikon Eclipse Ti-U fluorescent microscope equipped with a Spot RT3 camera using a 60× objective lens. At least 25 cells were randomly selected and analyzed. Comet attributes were analyzed using ImageJ 1.43f software.

### Analysis of Nuclear Fragmentation

2,000 cells/well were seeded in 96-well plates and cultured overnight before treatments. Following treatments, the cells were washed with 1 × PBS and incubated with 0.5 μg/ml Hoechst 33342 for 10 min. Nuclei were visualized by a Nikon Eclipse Ti-U fluorescence microscope equipped with a Spot RT3 camera using 4× and 20× objective lenses. Images were recorded using a 4× objective lens. Nuclei having condensed or fragmented apoptotic characteristics were quantified using the ImageJ software (NIH). For each treatment, at least 300 nuclei were evaluated.

### Analysis of Mitochondrial Morphology

Mitochondrial morphology of B16F10 cells transfected with pDsRed2-Mito either alone or in combination with pPARPGFPC1/N3 construct were analyzed by confocal microscopy. Confocal Z-stacks were acquired using an IMIC Andromeda system (Fondis Electronic) equipped with a 60× oil immersion objective (UPLAN 60× oil, 1.35NA, Olympus, Milan, Italy) at 488 and 561 nm excitation wave lengths using HC 525/39 and HC 615/20 (Semrock) emission filters. Length of mitochondria was determined by measuring 10 mitochondria per cell manually using the ImageJ software (NIH). In each sample, at least 20 cells were analyzed.

### Tetramethyl-Rhodamine Methyl Ester (TMRM) Time-Laps Fluorescence Imaging

Cells were incubated in 10 nM TMRM [dissolved in Hank’s Balanced Salt Solution (HBSS)] supplemented with 10 mM 4-(2-hydroxyethyl)-1-piperazineethanesulfonic acid (HEPES), in the presence of 1 μM P-glycoprotein inhibitor cyclosporine H in 5% CO_2_ atmosphere at 37°C for 30 min as previously described in Frezza et al. (2006). Sequential images of TMRM fluorescence were acquired every 60 s using the aforementioned IMIC Andromeda system for 30 min. As for depolarization control, 2 μM oligomycin and 2.5 μM carbonyl cyanide p-trifluoro-methoxyphenyl hydrazone (FCCP) were added at 5 and 25 min post-treatment, respectively. Analyses of the TMRM fluorescence of the mitochondrial regions of interest were carried out using the ImageJ software (NIH). Data are expressed as average ±SEM of at least 3 independent experiments.

### Immunoblot Analysis

B16F10 cells were harvested in cold RIPA lysis buffer complemented with 1% protease inhibitor cocktail and 10% Phos-stop phosphatase inhibitor mixture (Roche, Sigma-Aldrich S.r.l., Milan, Italy), incubated on ice for 30 min and centrifuged at 10,000 *g*, at 4°C for 15 min. Protein concentration was determined using Bradford reagent (Bio-Rad Laboratories S.r.l., Milan, Italy). Proteins (20 μg/lane) were separated on Tris-acetate 3–8% or Bis-Tris 4–12% (NuPAGE, Life Technologies, Milan, Italy) polyacrylamide gels (Life Technologies, Milan, Italy) and transferred to PVDF membranes (Merck, Sigma-Aldrich S.r.l., Milan, Italy) that were blocked in 5% Bovine Serum Albumin (BSA) diluted in 0.1% Tween-20 containing tris-buffered saline (TBST) at room temperature for 1 h. Primary antibodies against OPA1 (Becton Dickinson Italia S.r.l., Milan, Italy, 1:1000), β-Actin (Sigma-Aldrich S.r.l. Milan, Italy 1:10 000), PAR (Santa Cruz Biotechnology, 1:500) were incubated in 5% BSA containing TBST. Horseradish peroxidase-conjugated anti-rabbit, anti-mouse or anti-rat (Bio-Rad Laboratories S.r.l., Milan, Italy) secondary antibodies were diluted in 1:3000 in 5% milk containing TBST and membranes were incubated at room temperature for 1 h. Peroxidase labeling was visualized using enhanced chemiluminescence substrate (Life Technologies, Pierce, Milan, Italy) and detected by an Image Quant mini Luminescent Image Analyzer 4000 (GE Healthcare Italia S.r.l., Milan, Italy).

### Statistical Analysis

Data were analyzed by one-way ANOVA with Tukey *post hoc* comparison tests with alpha = 0.05; *n* ≥ 3. Significance was expressed as ^∗^*p* < 0.05, ^∗∗^*p* < 0.01, ^∗∗∗^*p* < 0.001.

## Results

### Effect of PJ34 and Cisplatin or Temozolomide on Viability, Colony Formation and Nuclear Fragmentation of B16F10 Melanoma Cells

**FIGURE 1:**
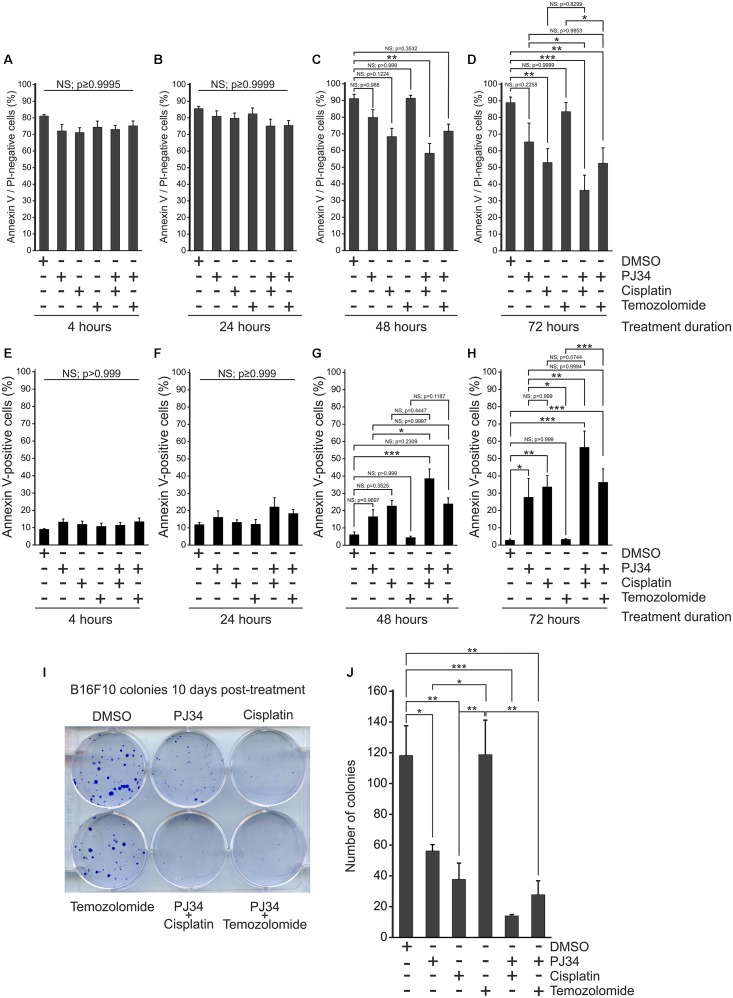
Effect of PJ34 in combination with cisplatin or temozolomide on cytotoxicity and colony formation in B16F10 melanoma cells. Cells were treated as indicated in the figure, labeled with Annexin V^FITC^ (AV) and propidium iodide (PI) and subjected to flow cytometry. AV and PI double-negative live **(A–D)** as well as AV positive apoptotic cells **(E–H)** are presented as % of all cells after 4 **(A,E)**, 24 **(B,F)**, 48 **(C,G)**, and 72 **(D,H)** hours of treatment. In a parallel experiment, B16F10 cells were treated as indicated in the figure and stained with trypan blue after 10 days of incubation. Representative image **(I)** and quantitative assessment **(J)** of colony formation is presented. Values are expressed as mean + SEM, *N* = 3, ^∗^*p* < 0.05, ^∗∗^*p* < 0.01, ^∗∗∗^*p* < 0.001; NS, non-significant.

It has been reported in various tumor models that cisplatin and temozolomide have enhanced cytotoxicity when used in combination with pharmacologic PARP inhibitors (Calabrese et al., 2004; Donawho et al., 2007). In order to investigate the putative combined effects of PJ34 and cisplatin or temozolomide on cellular viability, we performed flow cytometry using Annexin-V^FITC^ and propidium iodide labeling in B16F10 melanoma cells (Figure 1A–H and Supplementary Figure S1). We found that neither the alkylating agents nor PJ34 showed cytotoxicity in B16F10 cultures within the first 24 h of treatment (Figure 1A,B). At 48 h post-treatment, PJ34 developed a mild, although statistical not significant, cytotoxicity (Figure 1C) that was further increased at 72 h post-treatment (Figure 1D). A similar trend was observed with cisplatin that significantly decreased the viability at 72 h post-treatment (Figure 1D). This effect, however, became more pronounced when cisplatin was used in combination with PJ34 for 48 and 72 h (Figure 1C,D). In contrast to cisplatin, temozolomide did not induce cytotoxicity in B16F10 cultures (Figure 1A;D). In combination with PJ34, however, decreased viability was detected 72 h post-treatment, although it was not statistically significant compared to PJ34 alone (Figure 1D).

**FIGURE 2:**
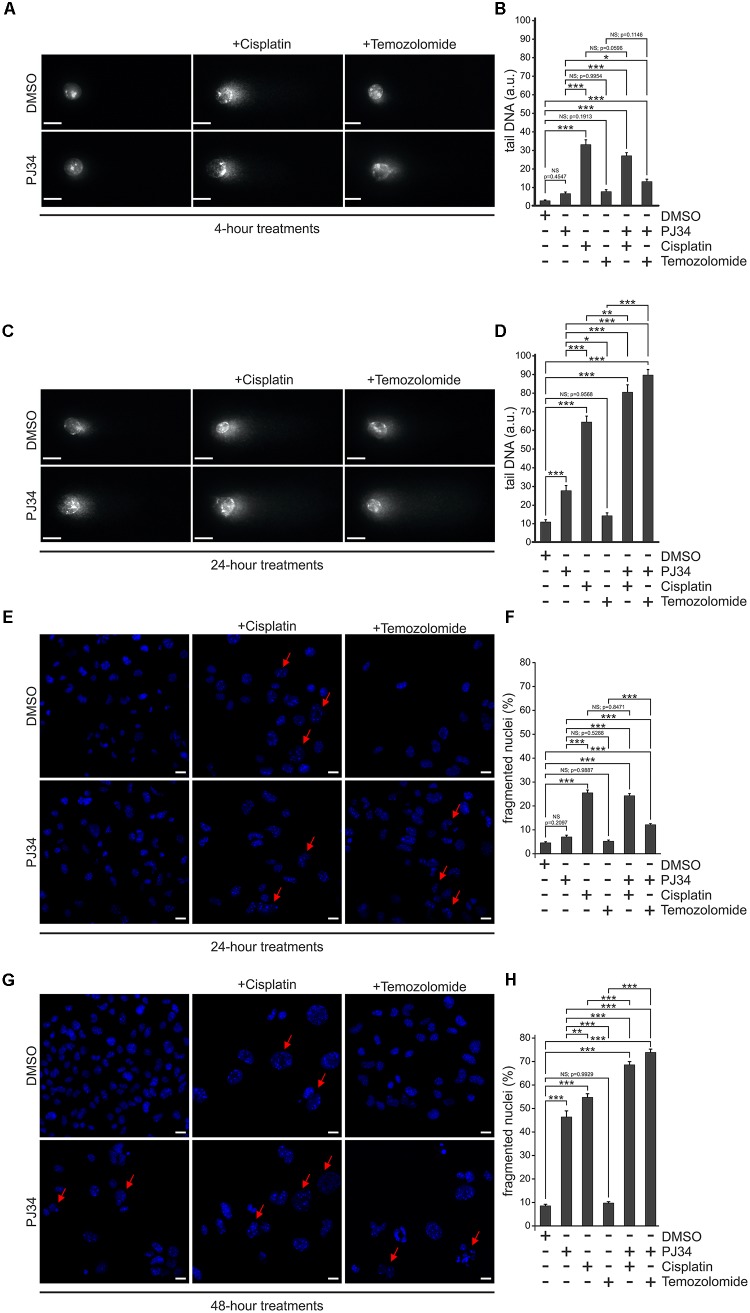
Effect of PJ34 on cisplatin and temozolomide-induced cell death and nuclear fragmentation in a B16F10 cells. Comet assay **(A–D)**. Cells were treated for 4 **(A,B)** or 24 **(C,D)** hours as indicated in the figure and a modified alkaline single cell gel electrophoresis was used to detect DNA damage. At least 25 cells per treatment group were randomly selected and analyzed. Comet attributes (tail DNA) were analyzed using ImageJ 1.43f software. Results of three independent experiments are presented as representative images **(A,C)** and bar diagrams **(B,D)** of mean + SEM tail DNA values expressed in arbitrary units (a.u.). Nuclear fragmentation **(E–H)**. Cells were treated as indicated in the figure for 24 **(E,F)** and 48 **(G,H)** hours and stained with Hoechst 33342 post-treatment. Panel **(E,G)** shows representative micrographs recorded by confocal microscopy. Nuclear morphology was assessed by evaluating a minimum of 300 nuclei per sample using the ImageJ software. Panel **(F,H)** shows quantification of the nuclear morphology, measured by the frequency of fragmented nuclei. Arrows indicate Hoechst 33342 stained cells having condensed or fragmented apoptotic nuclei. Values are expressed as mean + SEM, *N* = 6, ^∗^*p* < 0.05, ^∗∗^*p* < 0.01, ^∗∗∗^*p* < 0.001; NS, non-significant. Scale bar 20 μm.

Over the first 4 h of treatment, the proportion of apoptotic (Annexin V positive) and necrotic (propidium iodide positive) species were not affected by any of the treatments (Figure 1E and Supplementary Figure S1). After 24 h, apoptosis tended to dominate (Figure 1F), although this effect did not reach the level of statistical significance. After 48 h, co-treatment with PJ34 and cisplatin caused significant increase in the abundance of Annexin V-positive cells (Figure 2G). 72 h post-exposure, all substances but temozolomide increased apoptosis significantly; in combination in a more pronounced way than alone (Figure 1H). We did not observe any significant change in necrosis (Supplementary Figure S1).

In order to evaluate the cytostatic effects of PJ34, cisplatin and temozolomide on B16F10 melanoma cells, we performed colony formation assays to observe cellular proliferation capacity (Figure 1I,J). We found that, unlike temozolomide, both PJ34 and cisplatin decreased colony formation in B16F10 cultures. This effect was more evident in cisplatin-treated cultures where the number of colonies was reduced by more than 60% at 10 days post-treatment. Temozolomide alone did not affect B16F10 colony formation, however, it reduced colony formation in combination with PJ34 moderately (Figure 1I,J).

In order to study the potential underlying mechanisms, we analyzed the DNA-damaging effects of the compounds used. B16F10 cells were exposed to cisplatin, temozolomide or PJ34 in different combinations and alkaline single cell gel electrophoresis assay was performed on at least 25 randomly selected cells in each treatment cohorts. We found that all compounds examined triggered DNA fragmentation (Figure 2A–D). Temozolomide-treated cells accumulated DNA breaks in comparable extent to that of PJ34 but they were less effective than cisplatin alone. Combination of the pharmacologic PARP inhibition with cisplatin or temozolomide, however, led to increased number of persisting DNA-breaks and this augmenting effect of PJ34 was more pronounced in combination with temozolomide. These data are in accordance with our cytotoxicity and colony formation assay results and support the idea of the existence of a synergistic effect between PARP inhibition and alkylating compounds in B16F10 cells.

In order to confirm the presence of elevated apoptosis, we quantified the number of apoptotic nuclei in B16F10 cells treated with cisplatin, temozolomide either alone or in combination with PJ34 (Figure 2E–H). We found that, unlike temozolomide and PJ34, cisplatin triggered apoptotic nuclear morphology promptly in B16F10 cells. Surpisingly, this effect was not increased any further by the use of PJ34 during the first 24 h of the treatments (Figure 2F). In contrast, combinatorial treatment with PJ34 and temozolomide resulted in significantly more apoptotic nuclei than that of the standalone temozolomide treatment (Figure 2F). More interestingly, 48 h post-treatment, PARP inhibition increased the number of apoptotic nuclei, an effect that was more pronounced in combination with cisplatin (Figure 2H).

Taken together, flow cytometry and nuclear morphology data indicate distinct kinetic of the cisplatin- and temozolomide-induced cytotoxicity in B16F10 cells. Unlike cisplatin that provoke a prominent early apoptotic response, the cytotoxic effect of temozolomide unfolds gradually. PJ34-mediated pharmacologic inhibition of PAPR-1 enhances the cytotoxic effect of both cisplatin and temozolomide and this effect was accompanied by hallmarks of apoptosis including elevated Annexin V binding and apoptotic nuclear morphology. The augmenting effect of PJ34, however, develops slower and requires 48 h to become significant.

### Effect of PJ34 and Cisplatin or Temozolomide on Mitochondrial Fragmentation in B16F10 Cells

**FIGURE 3:**
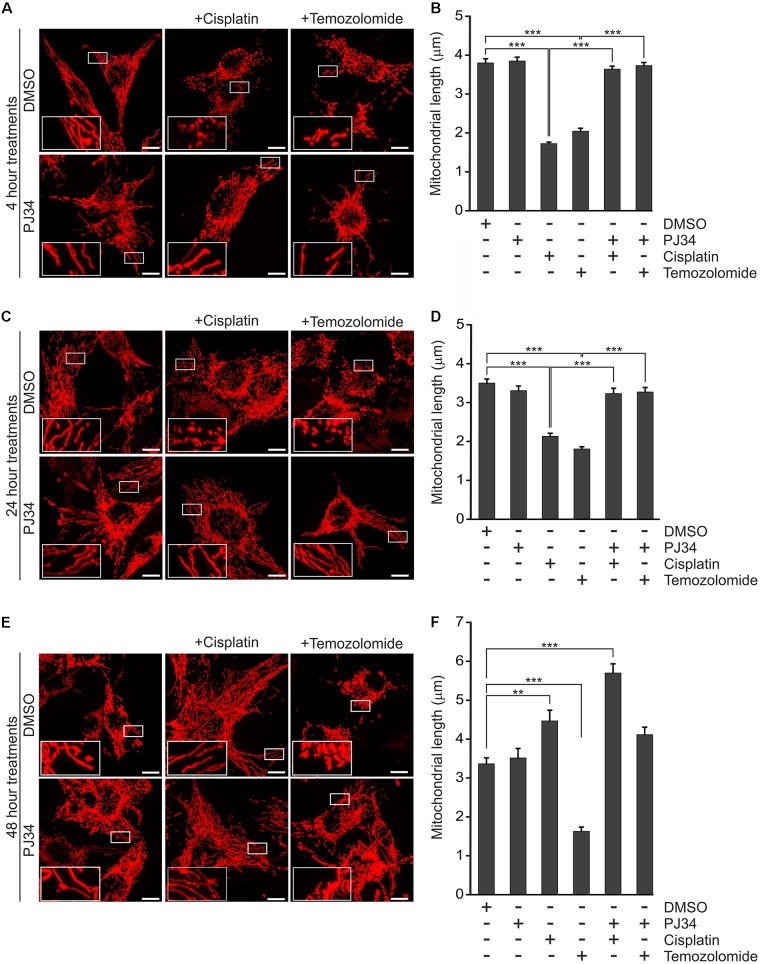
Effect of PJ34, cisplatin and temozolomide on mitochondrial fragmentation. B16F10^mtRFP^ cells were treated with cisplatin, temozolomide, PJ34 and their combinations. Representative reconstructions of confocal *z*-stacks are shown after 4 **(A)** 24 **(C)**, and 48 **(E)** hours post-treatment. Panel **(B,D,F)** shows quantification of the mean length of mitochondria at 4, 24-, and 48-h post-treatments, respectively. Values are expressed as mean + SEM, *N* = 3, ^∗^*p* < 0.05, ^∗∗^*p* < 0.01, ^∗∗∗^*p* < 0.001, Scale bar: 10 μm.

A number of studies proposed a model that the cytotoxic effect of cisplatin is, at least in part, mediated by mitochondria (Gordon and Gattone, 1986; Rosen et al., 1992; Olivero et al., 1995; Yang et al., 2006). Interestingly, mitochondria have also been suggested as target of pharmacologic PARP inhibition raising the question whether mitochondrial events are involved in the cytotoxic and cytostatic effects of PJ34, cisplatin, and temozolomide in the B16F10 melanoma model (Cseh et al., 2017). In order to investigate this hypothesis, we studied the integrity of the mitochondrial network using B16F10 cells expressing a mitochondria-directed red fluorescent protein (B16F10^mtRFP^) (Figure 3). In accordance with literature data from other models (Choi et al., 2015), elevated mitochondrial fragmentation was observed in melanoma cells treated with cisplatin as early as 4 h post-treatment (Figure 3A,B). A similarly swift disruption of the mitochondrial network was observed in temozolomide-treated B16F10 cells (Figure 3A,B). In contrast, pharmacologic PARP inhibition by PJ34 did not affect mitochondrial morphology. Moreover, PJ34 attenuated the mitochondrial effects of cisplatin and temozolomide maintaining the hyperfused mitochondrial phenotype when used in conjunction with the cytostatic compounds. This mitochondrial effect was found to be stable for at least 48 h post-treatment (Figure 3E,F).

**FIGURE 4:**
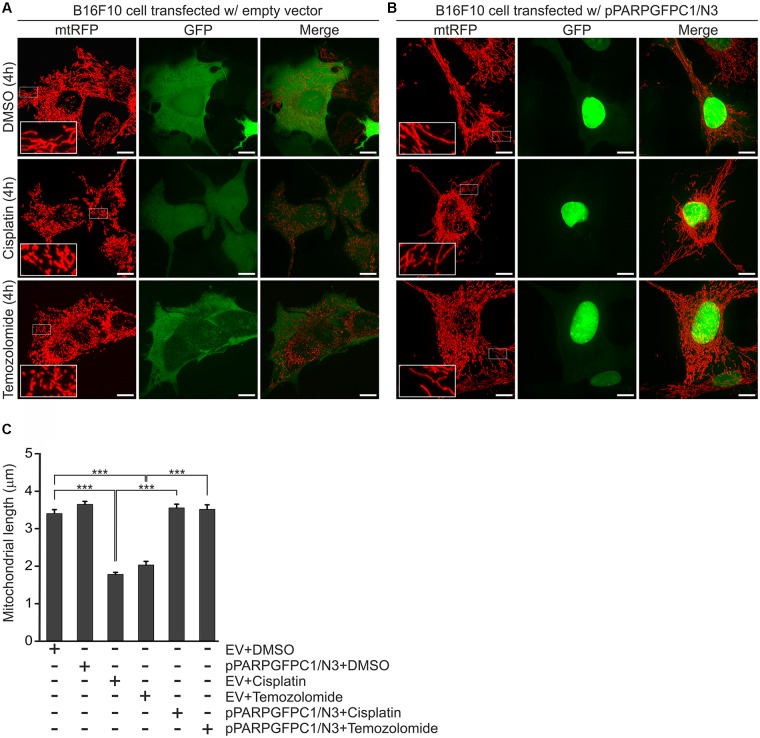
Effect of PARP^DN^, cisplatin and temozolomide on mitochondrial fragmentation. B16F10 cells were co-transfected with either mtRFP and an empty vector (EV) **(A,C)** or mtRFP and pPARPGFPC1/N3 **(B,C)**, treated as indicated in the figure for 4 h and the lengths of mitochondria were quantified. Panel **(A,B)** show representative reconstructions of confocal *z*-stacks while **(C)** displays the quantification of the mean length of mitochondria at 4-h post-treatments. Values are expressed as mean + SEM, *N* = 3, ^∗^*p* < 0.05, ^∗∗^*p* < 0.01, ^∗∗∗^*p* < 0.001. Scale bar: 10 μm.

In order to confirm if the observed mitochondrial effects of PJ34 are mediated by the blockade of PARylation activity, B16F10^mtRFP^ cells were transfected with a GFP-tagged peptide spanning the N-terminal DNA-binding domain of PARP-1 (PARP^DN^) (Figure 4A–C). PARP^DN^ acts as a dominant negative PARP competing with endogenous PARPs for PARP recognition loci without exerting PARylation activity. PARP^DN^-transfected B16F10^mtRFP^ cells were treated with cisplatin or temozolomide for 4 h and the mitochondrial structures were assessed as above (Figure 4A–C). We found that ectopic expression of PARP^DN^ did not affect morphology of the mitochondrial network in the absence of alkylating agents. Down-regulation of the endogenous PARP activity by PARP^DN^, however, attenuated the mitochondrial fragmentation triggered by both cisplatin and temozolomide supporting the concept that the mitochondrial effects of PJ34 are mediated by the reduction of endogenous poly(ADP-ribosyl)ation.

In order to investigate the potential underlying mechanisms of the PJ34 protective effects on mitochondrial morphology, we studied the nuclear encoded Dynamin-like 120 kDa mitochondrial protein OPA1, one of the key regulators of mitochondrial dynamics (Ishihara et al., 2006). OPA1 is expressed as long and short isoforms (L-OPA1 and S-OPA1, respectively) in mitochondria and both are believed to be critical for proper mitochondrial fusion, integrity of *cristae* junctions (Frezza et al., 2006; Song et al., 2007) and susceptibility to Cytochrome C release to engage apoptosis (Frezza et al., 2006). Since balanced expression of OPA1 isoforms promotes mitochondrial fusion, we raised the question whether the PJ34-mediated blockade of PARylation impacts the ratio of the OPA1 isoforms.

**FIGURE 5:**
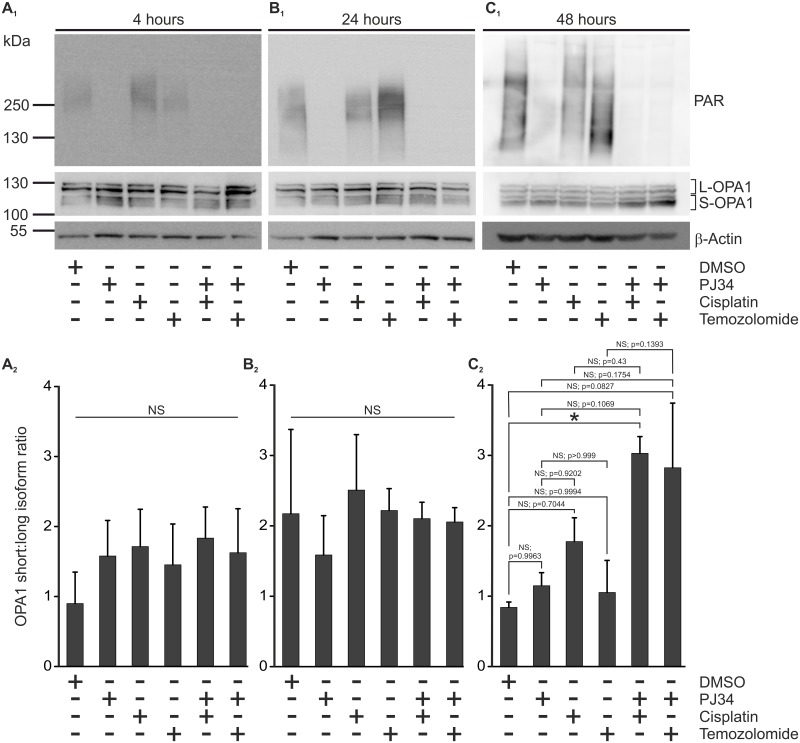
Effect of PJ34 and cisplatin or temozolomide on ratio of short per long OPA1 isoforms in B16F10 cells. Panel **(A_1_,B_1_,C_1_)** shows representative immunoblots of PAR and OPA1 from cells treated as indicated in the figure. S-OPA1 and L-OPA1 indicate its short and long isoforms, respectively. β-Actin was used for loading control. Panel **(A_2_,B_2_,C_2_)** display densitometry results of corresponding immunoblots. Values are expressed as mean + SEM, *N* = 3, ^∗^*p* < 0.05, ^∗∗^*p* < 0.01, ^∗∗∗^*p* < 0.001; NS, non-significant.

To address this question, we performed immunoblot analyses for OPA1 isolated from B16F10 cells treated with cisplatin or temozolomide in the presence or absence of PJ34. To monitor the efficacy of the PJ34 treatment, cell lysates were also tested for the presence of PAR polymers (Figure 5). We found no differential expression of either the short or the L-OPA1 in first 24 h post-treatment (Figure 5A_1_,A_2_,B_1_,B_2_). In contrast, an increase of S-OPA1 was detected in melanoma cells treated with PJ34 in combination with cisplatin for 48 h (Figure 5C_1_,C_2_). A similar trend was observed for PJ34 in combination with temozolomide, although the densitometry data was not statistically significant. Taken together, marked accumulation of S-OPA1 was only detected in cells treated with both PJ34 and cisplatin, and to a lesser extent temozolomide, for 48 h when combination treatments rather favors fusion of mitochondria (Figure 3E,F). Since accumulation of S-OPA1 does not correlate with the observed morphological deterioration of the mitochondrial network, these data suggest that the cisplatin-, temozolomide- and PJ34-related morphological mitochondrial effects are not mediated by OPA1.

### Effect of PJ34 and Cisplatin or Temozolomide on Mitochondrial Membrane Potential

**FIGURE 6:**
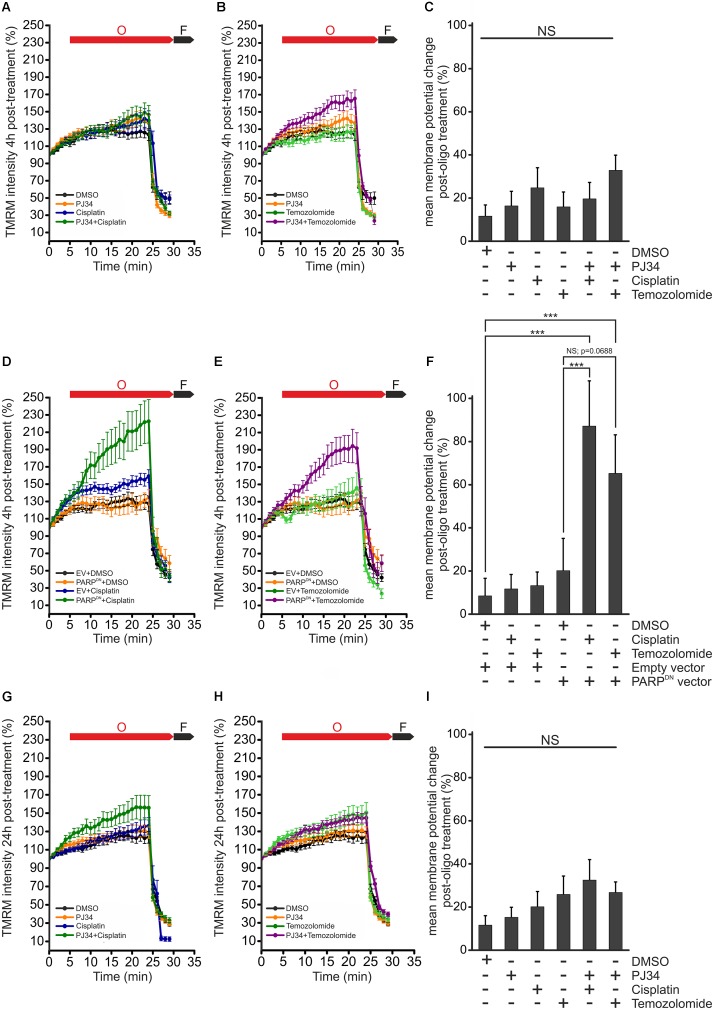
Effect of PJ34 and cisplatin or temozolomide on mitochondrial membrane potential. Wild type **(A–C,G–I)** and PARP^DN^-expressing B16F10 cells **(D–F)** were treated for 4 h **(A–F)** and 24 h **(G–I)**, incubated with TMRM and the red fluorescence correlating with the mitochondrial transmembrane potential was monitored for 30 min. In each experiment, cells were treated with 2 μM oligomycin and 2.5 μM FCCP, 5 and 25 min after the start of measurements, respectively. O, oligomycin; F, FCCP. Panels **(C,F,I)** shows percent change in mitochondrial membrane potential post-oligomycin treatments. Data are expressed as mean + SEM, *N* = 3, ^∗^*p* < 0.05, ^∗∗^*p* < 0.01, ^∗∗∗^*p* < 0.001; NS, non-significant.

Mitochondrial morphology is believed to be intimately connected to mitochondrial function and metabolism. Indeed, it was reported that reduction of the electrochemical potential across the inner mitochondrial membrane is accompanied by cleavage of L-OPA1 leading to accumulation of S-OPA1 and mitochondrial fragmentation (Duvezin-Caubet et al., 2006; Ishihara et al., 2006; Song et al., 2007). Since human melanoma xenografts have been reported to have one of the highest oxygen consumption rates among tumors, it is believed that an intact mitochondrial metabolism is critical for the survival of melanoma cells (Kallinowski et al., 1989; Barbi de Moura et al., 2012). In order to investigate whether the cisplatin, temozolomide and PJ34-mediated effects on mitochondrial morphology are associated with functional alterations, we assessed the mitochondrial respiratory chain by measuring the mitochondrial membrane potential (ψ_m_) (Figure 6). In order to study the functionality of the mitochondrial respiratory chain, membrane potential was monitored in cells treated with the ATP synthase inhibitor oligomycin. For positive control, depolarization of the mitochondrial inner membrane was triggered by the mitochondrial uncoupler carbonyl cyanide p-trifluoro-methoxyphenyl hydrazone (FCCP). We found that neither cisplatin nor temozolomide affected the ψ_m_. PJ34 did not influence the mitochondrial membrane potential either. Interestingly, when PJ34 was used in combination with the cytostatic agents, we did not detect the collapse of the mitochondrial membrane potential either (Figure 6A–C,G–I). Instead, a slight, although statistically not significant, trend of membrane hyperpolarization was seen. Even more surprisingly, this trend unfolded more promptly in the presence of temozolomide compared to cisplatin (Figure 6A–C). In order to confirm the PARylation dependency of the observed mitochondrial membrane hyperpolarization, we repeated the above experiments using cells transfected with a dominant negative mutant of PARP-1 (PARP^DN^). Expression of PARP^DN^ alone did not influence the mitochondrial membrane potential. In contrast, however, robustly elevated mitochondrial membrane potential was measured in the PARP^DN^ expressing B16F10 cells treated with cisplatin or temozolomide (Figure 6D–F). These data suggest that the cytotoxic effect observed in B16F10 cells exposed to both PJ34 and the cytostatic agents is not mediated by the collapse of the mitochondrial membrane potential.

To further investigate the potential interplay between the PJ34-mediated enhancement of the alkylating agents cytotoxic effects, we also tested functionality of the mitochondrial NADPH reducing system performing MTT assays in cells treated with combinations of PJ34, cisplatin, and temozolomide. In accordance with our observations on the mitochondrial membrane potential, however, we did not find any difference in the activity of the mitochondrial NADPH reductases in the presence of PJ34 (Supplementary Figure S2). Taken together, our data suggest that the mitochondrial and cytotoxic effects of PJ34 do not correlate and reflects on parallel but independent molecular mechanisms.

## Discussion

Platinum agent-based chemotherapy has been in the clinical practice for decades in various types of human neoplasms. Traditionally, it is believed that their mechanism of action is based on the formation of nuclear platinum-DNA adducts that lead to DNA-damage, cell-cycle arrest and, eventually, apoptosis (Rosenberg et al., 1965; Takahara et al., 1995). Accordingly, they likely provoke an excess rate of the cellular DNA-damage response that may also contribute to their cytotoxic effect via, at least in part, PARP-mediated functions (Kelland, 2007; Thomadaki and Scorilas, 2007). The idea of the interplay between PARylation and cisplatin has been proposed in the early 1990s on the basis of the central role of PARP-1 in the nuclear DNA repair mechanisms termed DNA damage response (Burkle et al., 1993).

This concept is in accordance with observations on PARP expression in human neoplasms that show elevated *PARP-1* expressions in a wide range of human cancers including high-grade astrocytomas, colorectal carcinomas, hepatocellular carcinomas or malignant breast lesions (Rojo et al., 2011; Li et al., 2017; Murnyák et al., 2017; Dörsam et al., 2018). These studies have also pointed out that increased PARP-1 levels are, apparently, associated with high-grade tumors and show inverse correlation with patient survival fueling the idea of the use of PARP-1 as a prognostic marker and therapeutic target (reviewed in Cseh et al., 2017).

The finding that PARP-1 promotes human primary melanocyte proliferation in a PARylation-independent manner mediated by the induction of the melanocyte-lineage survival oncogene *MITF* suggest a universal role of PARP-1 in carcinogenesis supporting the concept of PARP inhibition as an anti-cancer modality (Choi et al., 2017). Indeed, PARP inhibition efficiently reduced the metastasizing capacity of melanoma cells in murine models (Rodriguez et al., 2013). The potential use of PARP inhibition in human melanomas has been further supported by a recent retrospective cohort study of 66 patients with metastatic melanoma treated with conventional chemotherapy using DNA alkylating compounds (Abecassis et al., 2019). Data evaluation revealed that the response to conventional chemotherapy inversely correlates with the expression of the endogenous PARP-1 variant carrying a single nucleotide polymorphism termed rs1805407 (SNP rs1805407). The observation that the use of ABT-888 and olaparib, two well-known PARP-inhibitors, improved the efficacy of chemotherapeutics on cancer cells carrying SNP rs1805407 suggests that this variant is associated with higher *PARP-1* expression and supports the idea of potential synergism between conventional therapeutics and pharmacologic PARP inhibition in the treatment of melanoma.

In the present study, we investigated the effects of two alkylating agents, cisplatin and temozolomide, commonly used in melanoma treatment and the PARP inhibitor compound PJ34 using the *in vitro* B16F10 melanoma model. We found a marked difference between cisplatin and temozolomide toxicity on B16F10 cells. Cisplatin exerted a slowly developing cytotoxicity that was statistically significant 72 h post-treatment. In contrast, temozolomide was found to be inefficient in provoking significant cell death in B16F10 cultures within the timeframe investigated. However, PJ34 potentiated the cytotoxicity of both cisplatin and temozolomide, the combined effect exceeded that of the individual compound and this effect of PJ34 was more pronounced on temozolomide. Similar observations were made on nuclear fragmentation, the ratio of apoptotic cells and inhibition of colony formation. Our findings are in agreement with those of others on combination of PARP inhibition and temozolomide (Erice et al., 2015).

Although the underlying mechanism is still to be elucidated, the observed differences between the synergistic effect of PARP-inhibition in the presence of cisplatin or temozolomide may be accounted for their distinct mechanism of action. Indeed, cisplatin is believed to inhibit proliferation by crosslinking DNA and induction of DNA breaks eventually leading to, predominantly, apoptosis (Prestayko et al., 1979). In contrast, temozolomide alkylates DNA that is repaired by the O^6^-alkylguanine DNA alkyl transferase (MGMT). This enzyme is expressed in a number of cancer cells including the B16F10 melanoma model (Hau et al., 2007; Shih et al., 2016). These data together with our own and others observations on the efficacy of PARP inhibition in combination with distinct chemotherapeutics suggest that the histological background of the target cells should be considered critical in the design of anti-cancer strategies applying PARP inhibitors.

Human melanoma xenografts have been reported to show extremely high oxygen consumption suggesting their particular dependency on the intact mitochondrial metabolism (Kallinowski et al., 1989; Barbi de Moura et al., 2012). Interestingly, intense research on the mechanism of action of cisplatin has proposed mitochondrial targets as alternative effectors of cytotoxicity as well (Cullen et al., 2007). Indeed, mitochondrial swelling, loss of *cristae* and disruption of the outer mitochondrial membrane were all observed in cisplatin-treated cells (Rosen et al., 1992). In accordance with these reports, we found extensive and robust disintegration of the mitochondrial network preceding the onset of cytotoxicity in cisplatin-treated B16F10 cells. A similar but more pronounced effect was observed in temozolomide-treated cells despite the limited cytotoxicity of this compound in the melanoma cultures examined. This finding suggests that unlike the nuclear ones, mitochondrial effect of temozolomide is not limited by MGMT activity. In agreement with previous reports (Orsucci et al., 2008; Fan et al., 2017; Tapodi et al., 2018), we found that PARP inhibition did not affect the mitochondrial network morphology. Moreover, PJ34 was found to preserve the mitochondrial network integrity in B16F10 cells treated when combined with either cisplatin or temozolomide.

One regulator of mitochondrial network dynamics is OPA1, a large mitochondrial dynamin like GTPase that facilitates fusion of mitochondria (Song et al., 2007). Although the underlying mechanisms are still not fully understood, OPA1 exerts its fusion-promoting effects on mitochondria via the tightly regulated equilibrium of long and short OPA1 isoforms (Del Dotto et al., 2017). Oligomeric complexes made of the S- and L-OPA1 contribute to the preservation of the mitochondrial *cristae* structures and, therefore, physiologic mitochondrial morphology (Frezza et al., 2006). In response to various stimuli, including collapsed membrane potential across the inner mitochondrial membrane, depleted mitochondrial ATP levels or pro-apoptotic signals, L-OPA1 is cleaved by proteases like Yme1L and OMA1 (Song et al., 2007; Anand et al., 2014) leading to the decomposition of OPA1 oligomers and suppression of fused mitochondrial morphology (Duvezin-Caubet et al., 2006; Ishihara et al., 2006; Guillery et al., 2008; Ehses et al., 2009). Interestingly, in B16F10 cells exposed to both PJ34 and the cytostatic agents examined, we detected a disturbed equilibrium of the OPA1 isoforms 48 h post-treatment. However, although this correlates to the onset of the cytotoxic effects of the combinatorial treatments, dysregulation of the OPA equilibrium was not manifested in corresponding morphological alterations of the mitochondrial network. These data suggest that if OPA1 contributes to the PJ34-augmented cytotoxic effects of cisplatin and temozolomide, it is mediated independently of its function in the regulation of mitochondrial morphology. Thus, whether the accumulation of S-OPA1 plays a functional role in the potentiating effect of pharmacologic PARP inhibition upon the use of alkylating agents needs further investigations.

It is widely accepted that fragmentation of mitochondria is a sign of cellular stress while fusion is usually present in cells with balanced metabolic homeostasis. Interestingly, in our experiments, the PJ34-mediated inhibition of mitochondrial fission did not rescue cells from cell death suggesting independent effects of PJ34 on viability and mitochondrial morphology in B16F10 cells. This concept is, apparently, supported by our observations that the PJ34-enhanced cytotoxicity is not accompanied by the collapse of the mitochondrial inner membrane potential, a hallmark of the failure of mitochondrial metabolism. Moreover, by using a dominant negative mutant PARP, we could also demonstrate that reduction of the cellular PARylation, leads to hyperpolarization in mitochondria, suggesting the existence of nuclear and mitochondrial PARylation-dependent mechanisms with distinct effects on cell survival.

Since the PJ34-facilitated cytotoxicity is preceded by pro-survival mitochondrial effects like the maintained mitochondrial fusion and elevated mitochondrial membrane potential, one can also speculate that sustained mitochondrial hyperpolarization results in mitochondrial damage through, for instance, enhanced ROS production raising the question if the observed accumulation of S-OPA1 is part of the clearance of damaged mitochondria via a late-onset mitochondrial fragmentation. This parallel with the attenuated DNA repair might lead to the augmented cytotoxic capacity of cisplatin and temozolomide.

## Conclusion

Our data suggest that the dominant, long-term effect of PARP inhibition when used in combination with alkylating cytostatics is enhancement of DNA damage, likely, by reducing repair activities. This synergism, eventually, supersedes the parallel observed mitochondria-protecting effects of PARP inhibition leading to increased nuclear fragmentation and eventually cell death.

**FIGURE 7:**
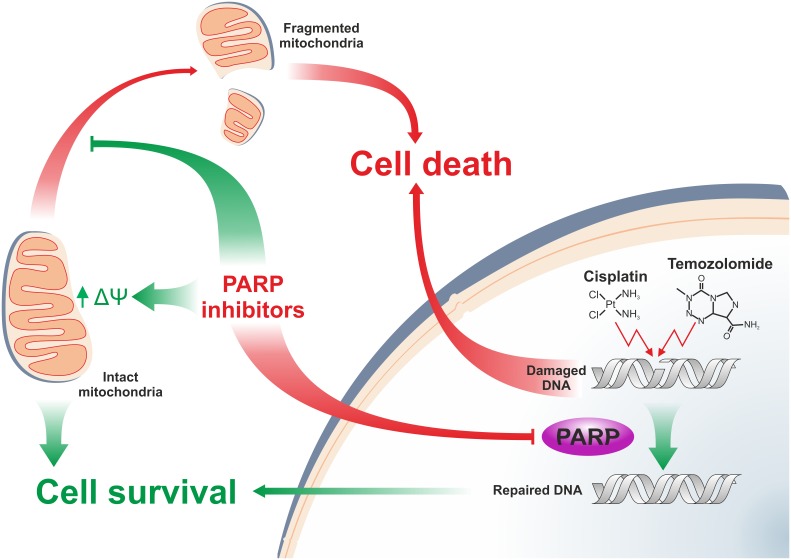
Effects of pharmacologic PARP inhibition in B16F10 melanoma cells. Schematic model on the synergism between the PARP inhibitor PJ34 and alkylating agent cisplatin and temozolomide. Arrows and blunt-end connector indicate catalyzed activation and inhibition, respectively. Green and red color refer to pro-survival and cell death-inducing activities, respectively.

Our data suggest, however, that the PARP inhibition-compromised DNA repair exacerbates the alkylating compound-mediated accumulation of nuclear DNA damage and nuclear fragmentation that, eventually, overcomes the pro-survival mitochondrial effects (Figure 7). Our model is in accordance with literature data on the overall effect of PARP modulation that is, seemingly, determined by the intricate relationship of the targeted molecular mechanisms (Sukhanova et al., 2016; Hocsak et al., 2017). The diverse, occasionally opposing effects of PARP inhibitors, even within the same model, however, underlines the importance of further, thorough evaluation of the use of these compounds in human pathologies.

## Author Contributions

AC designed and performed the experiments, evaluated data, and wrote the manuscript. ZF designed experiments, evaluated data, and prepared the manuscript. RQ-C and MS supervised TMRM experiments. AS performed alkaline single-cell gel electrophoresis and nuclear fragmentation experiments. KE contributed to the statistical analyses. FG reviewed the manuscript. LS and BS contributed equally to this work as supervisors.

## Conflict of Interest Statement

The authors declare that the research was conducted in the absence of any commercial or financial relationships that could be construed as a potential conflict of interest.

## Abbreviations

AKT/PKB: AKT/protein kinase B
ATF4: activating transcription factor 4
ATM: ataxia telangiectasia mutated
AV: annexin V
BRCA1/2: breast cancer type 1/2 susceptibility protein
BSA: bovine serum albumin
FCCP: *p*-trifluoro-methoxy-phenyl-hydrazone
FITC: fluorescein isothiocyanate
HBSS: Hank’s balanced salt solution
HEPES: 4-(2-hydroxyethyl)-1-piperazine-ethane sulfonic acid
L-OPA1: long isoforms of OPA1
MAP kinases: mitogen-activated protein kinase
MAPK: mitogen-activated protein kinase
MKP-1: mitogen-activated protein kinase phosphatase 1
mTOR: mammalian target of rapamycin
NAD^+^: nicotinamide-adenine-dinucleotide
NADPH: nicotinamide adenine dinucleotide phosphate
NEMO: NF-kappa-B essential modulator
OPA1: dynamin-like 120 kDa mitochondrial protein
PAR: Poly(ADP-ribose)
PAR: poly(ADP-ribose) polymers
PARP: Poly(ADP-ribose) polymerase
PI: propidium iodide
PI-3K-AKT: phosphatidylinositol-3-kinase and protein kinase B
S-OPA1: short isoforms of OPA1
TBST: Tween-20 containing tris-buffered saline
TMRM: tetramethyl-rhodamine methyl ester
