# Supplementary figures and images for: PARP Inhibitor PJ34 Protects Mitochondria and Induces DNA-Damage Mediated Apoptosis in Combination With Cisplatin or Temozolomide in B16F10 Melanoma Cells
Source: Front Physiol. 2019 May 7;10:538. doi: 10.3389/fphys.2019.00538 (PMC6514236; doi:10.3389/fphys.2019.00538)

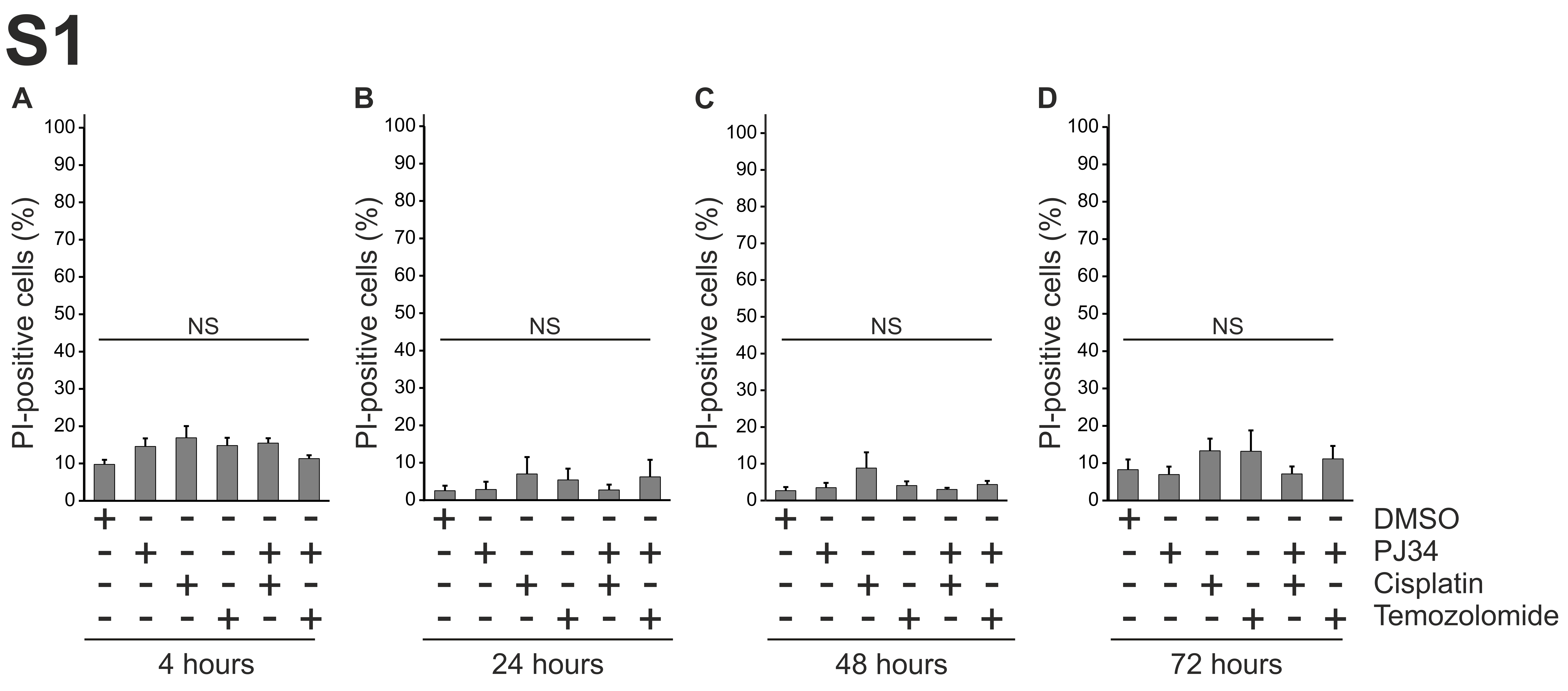

Supplement: Supplementary file 1 [file Image_1.TIF]

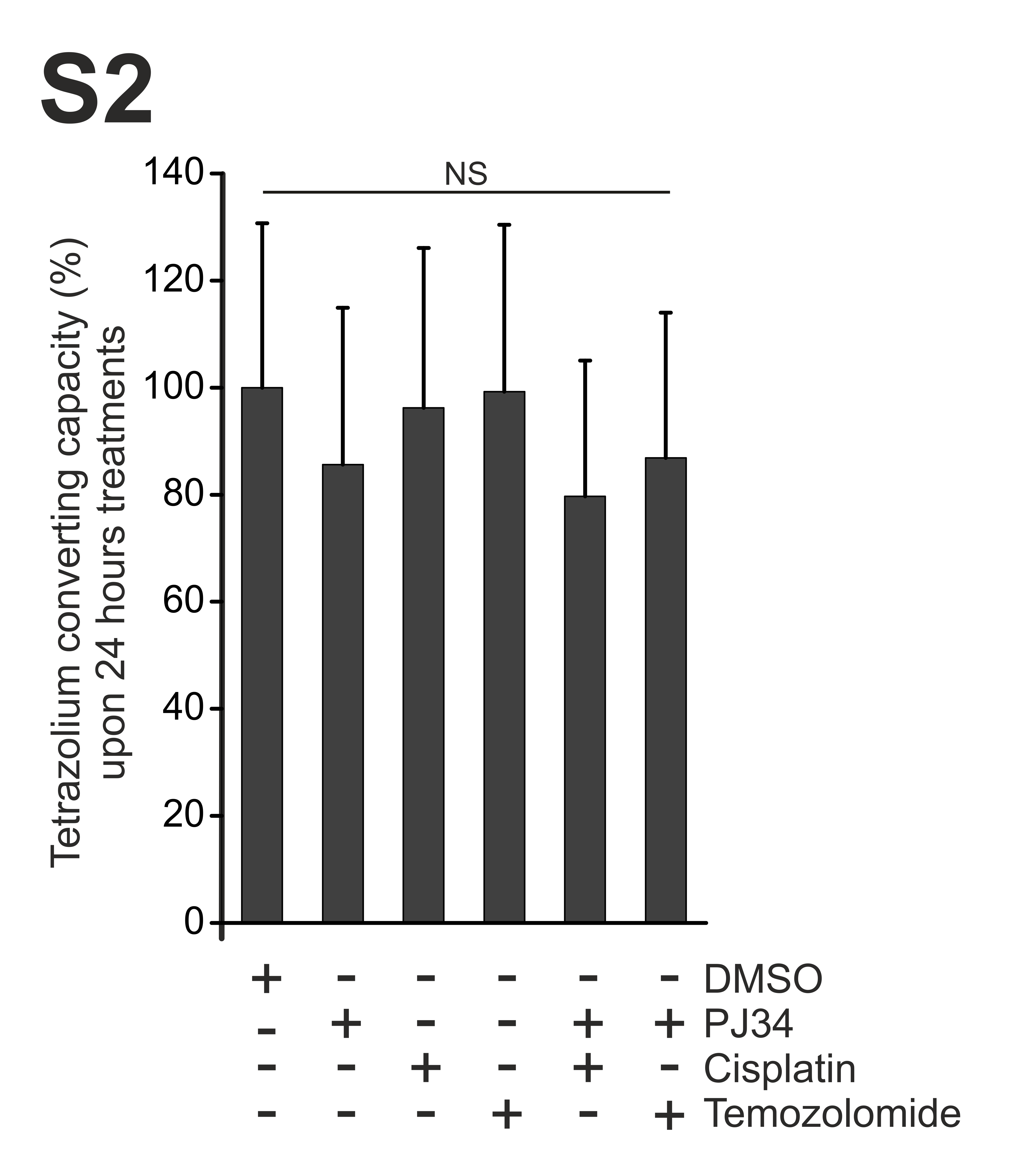

Supplement: Supplementary file 2 [file Image_2.TIF]
