# Supplementary material for: PARP Inhibitor PJ34 Protects Mitochondria and Induces DNA-Damage Mediated Apoptosis in Combination With Cisplatin or Temozolomide in B16F10 Melanoma Cells
Source: Front Physiol. 2019 May 7;10:538. doi: 10.3389/fphys.2019.00538 (PMC6514236; doi:10.3389/fphys.2019.00538)
Supplement: Supplementary file 3 [file Data_Sheet_1.pdf]

Supporting information

Supplementary Figure S1. PJ34 does not increase propidium iodide uptake of B16F10 cells. In order to investigate the combined effects of PJ34 and cisplatin or temozolomide on necrotic cell death, we performed flow cytometric viability assays using propidium iodide labeling in B16F10 melanoma cells. Cells were treated as indicated in the figure, labeled with propidium iodide (PI) and subjected to flow cytometry. We found no significant difference in PI staining. PI positive necrotic cells are presented as % of all cells after 4 (**A**), 24 (**B**), 48 (**C**) and 72 (**D**) hours of treatment. Values are expressed as mean+SEM of three independent experiments. *$p<0.05$; **$p<0.01$; ***$p<0.001$, NS=non-significant.

Supplementary Figure S2. PJ34 does not influence the NADPH reducing capacity of B16F10 cells. In order to test if the mitochondrial NADPH reducing capacity is involved in the observed cytotoxic effects, we performed MTT assays using B16F10 melanoma cells. Cells were treated as indicated in the figure for 24 hours. We found no significant difference in NADPH reducing capacity. Tetrazolium converting capacity of each group was expressed as ratio of DMSO-treated control sample. Values are expressed as mean+SEM of three independent experiments. *$p<0.05$; **$p<0.01$; ***$p<0.001$, NS=non-significant.
